# Transcriptome analysis of different life-history stages and screening of male-biased genes in *Daphnia sinensis*

**DOI:** 10.1186/s12864-022-08824-x

**Published:** 2022-08-13

**Authors:** Ziyan Wang, Feiyun Zhang, Qide Jin, Yeping Wang, Wenping Wang, Daogui Deng

**Affiliations:** College of Life Sciences, Huaibei Normal University, 100 Dongshan Road, Huaibei, Anhui China

**Keywords:** *Daphnia*, Life-history stages, Transcriptome, Male-biased genes, Differentially expressed genes

## Abstract

**Background:**

In the life history of *Daphnia*, the reproductive mode of parthenogenesis and sexual reproduction alternate in aquatic ecosystem, which are often affected by environmental and genetic factors. Recently, the sex-biased genes are of great significance for clarifying the origin and evolution of reproductive transformation and the molecular regulation mechanism of sex determination in *Daphnia*. Although some genes on reproductive transition of *Daphnia* had been researched, molecular mechanism on the maintenance of sexually dimorphic phenotypes of *Daphnia* are still not well known, including differentially expressed genes in different life-history stages.

**Results:**

In this study, four life-history stages of *Daphnia sinensis*, juvenile female (JF), parthenogenetic female (PF), sexual female (SF) and male (M), were performed for transcriptome, and male-biased genes were screened. A total of 110437 transcripts were obtained and assembled into 22996 unigenes. In the four life-history stages (JF, PF, SF and M), the number of unique unigenes is respectively 2863, 445, 437 and 586, and the number of common unigenes is 9708. The differentially expressed genes (DEGs) between male and other three female stages (M vs JF, M vs PF and M vs SF) were 4570, 4358 and 2855, respectively. GO gene enrichment analysis showed that the up-regulated genes in male were mainly enriched in hydrolase activity and peptidase activity. Thirty-six genes in male were significantly higher expression than in the three female stages, including one *Doublesex* (*Dsx*) gene, one laminin gene, five trypsin genes and one serine protease genes, and one chitin synthase gene and two chitinase genes.

**Conclusions:**

Our results showed that thirty-six candidate genes may be as the male-biased genes involving in the maintenance of sexually dimorphic phenotypes. This work will provide a reference for further exploring the functional genes related to sex differentiation in *Daphnia* species. Moreover, according to previous investigations, we thought that the expression level of functional genes may be related to the life-history stages of organisms, and may be also affected by different *Daphnia* species.

**Supplementary Information:**

The online version contains supplementary material available at 10.1186/s12864-022-08824-x.

## Background

The phenotypes of male and female individuals for one species usually show difference greatly [1]. This difference is often driven by genes on the sex chromosome, which showed a particularly strong sex bias expression tendency [2, 3]. Sex-biased genes of male and female fundamentally lead to the difference between male and female phenotypes [4]. Therefore, the sex-biased genes are great significance to clarify the origin and evolution of reproductive transformation and molecular regulation mechanism of sex differentiation.

In the life history of *Daphnia*, parthenogenesis and sexual reproduction often alternate in aquatic ecosystem, which is affected by environmental (e.g. food, predation, photoperiod) and genetic (e.g. genotype) factors together [5]. Under suitable environmental conditions, they only produce female offspring by parthenogenesis. However, when environmental conditions deteriorate (such as fish predation, food shortage and higher population density), *Daphnia* species will transfer from parthenogenesis to sexual reproduction, producing male and sexual female, and then mate and fertilize, forming resting eggs [6–9]. Resting eggs can survive in lake sediments for several decades [10], and then hatch and form new populations in suitable conditions. Some investigations have indicated that *Daphnia* is an ideal model organism in studying ecology, environmental toxicology and evolutionary biology [11, 12].

The sex maintenance and the switch of *Daphnia* species have affected by environmental changes, making *Daphnia* an interesting comparative system for the study of sex-biased and reproductive genes. Some study of *Daphnia pulex* revealed that 50% of assayed transcripts show some degree of sex-bias [1]. Among them, female-biased transcription is enriched for translation, metabolic and regulatory genes associated with life-history. Colbourne et al. (2011) showed the detailed genomes information of *D. pulex*, the amount of genomic and transcriptomic resources for the genus has markedly increased, which greatly promoted our understanding of sex-biased genes [13]. Zhang et al. (2016) constructed the genetic data sets of the genes expressed in a sexual female and a parthenongentic female of *Daphnia similoides*. The study showed the gene may have a crucial role in reproductive switching of *D. similoides*. Male-biased expression is enriched for cuticle and protease function [14]. Huylmans et al. (2016) and Molinier et al. (2018) analyzed the sex-biased genes between the female and male of *Daphnia galeata* and *Daphnia magna*, respectively [15, 16]. Moreover, the female-biased genes of *Daphnia sinensis* might contribute to maintaining rapid production of parthenogenetic females, and nutrient uptake for the growth of neonates [17]. Therefore, the sex-biased genes (including male-biased genes) might play a pivotal role in steering the life-history and reproduction processes in *Daphnia* species.

Although some male-biased genes in other species of *Daphnia* have been reported, it is still worth exploring whether these genes perform the same function in *D. sinensis. D. sinensis* is a typical *Daphnia* species that is widely distributed in inland fresh waters, particularly in eutrophic waters. Our goals are to compare the transcriptome of *D. sinensis* at four life history stages (i.e. juvenile female, parthenogenetic female, sexual female and male), and to explore the role of male-biased genes in male production and male phenotype maintenance. Our study will provide some necessary fundamental data for further research of sex-biased genes of *Daphnia* in future.

## Methods

### *D. sinensis *and* Tetradesmus obliquus* culture

*D. sinensis* employed in the experiment were collected from Lake Chaohu in Anhui Province, China. Parthenogenetic females were cultivated under a 12 h light /12 h dark photoperiod at 25 ± 1℃ with a light intensity of 2500 lx, and fed with 40 mg L^−1^ of *T. obliquus* (wet weight). *T. obliquus* was obtained from the Freshwater Algae Culture Collection at Institute of Hydrobiology, Chinese Academy of Sciences, and cultured in BG-11 medium at 25 ± 1℃ with a 12 h light/12 h dark photoperiod.

### Juvenile female (JF), parthenogenetic female (PF), sexual female (SF) and male (M) collection

In one experiment, 20 *D. sinensis* mothers were employed. Each mother was respectively cultured in a 50 ml beaker with 40 ml culture medium. The culture medium was replaced every two days. The culture medium was the filtered and aerated tap water. Offspring (birth time < 12 h) produced by *D. sinensis* mother were collected as juvenile female samples (JF), and each juvenile female sample was about 500 individuals. Juvenile female (JF) is female of *D. sinensis* within 12 h of birth**.** These neonates produced by the mother were removed in time from beakers during the experiment.

In another experiment, 60 *D. sinensis* mothers were employed. Each 10 mothers were placed in a 250 ml beaker with 200 ml culture medium, 6 replicates were conducted. The culture medium was replaced every two days. After 2–3 weeks, sexual females and males of *D. sinensis* could be observed when population density became higher. The parthenogenetic females (PF), sexual females (SF) and males (M) were selected under the microscope. Parthenogenetic female (PF) is female of *D. sinensis* carrying eggs for the first time; Sexual female (SF) is female of *D. sinensis* carrying resting eggs; Male is male of *D. sinensis* within 12 h of birth. About 50 parthenogenetic females and 50 sexual females were respectively collected as PF sample and SF sample. Male juveniles were collected and cultured in a 100 mL beaker with 80 mL culture medium for two weeks. Each male sample (M) contained about 60 male individuals.

JF, PF, SF and M samples were put into EP tubes, respectively. These samples were immediately frozen in liquid nitrogen and stored at -80℃. In this study, two replicates of JF, PF, SF and M samples were collected for transcriptome sequencing, respectively.

### RNA isolation and cDNA library construction

Total RNA was respectively extracted from samples at four life-history stages of *D. sinensis* (juvenile females, parthenogenetic females, sexual females and males) using TRIzol reagent. RNA degradation and contamination was monitored using 1% agarose gels. RNA purity was checked using the NanoPhotometer® spectrophotometer (IMPLEN, CA, USA). The RIN (RNA integrity number) value range of quality test is 5.8 to 6.6. RNA concentration was measured using Qubit® RNA Assay Kit in Qubit® 2.0 Flurometer (Life Technologies, CA, USA). RNA integrity was assessed using the RNA Nano 6000 Assay Kit of the Agilent Bioanalyzer 2100 system (Agilent Technologies, CA, USA).

A total of 1.5 µg RNA per sample was used as input material for the RNA sample preparations. Sequencing libraries were generated using NEBNext® Ultra™ RNA Library Prep Kit for Illumina® (NEB, USA) according to manufacturer’s recommendations, and index codes were added to attribute sequences to each sample. Briefly, mRNA was purified from total RNA using poly-T oligo-attached magnetic beads. Fragmentation was carried out using divalent cations under elevated temperature in NEBNext First Strand Synthesis Reaction Buffer (5X). First strand cDNA was synthesized using random hexamer primer and M-MuLV reverse transcriptase (RNase H^−^). Second strand cDNA synthesis was subsequently performed using DNA polymerase I and RNase H. Remaining overhangs were converted into blunt ends via exonuclease/polymerase activities. After adenylation of 3’ ends of DNA fragments, NEBNext Adaptor with hairpin loop structure was ligated to prepare for hybridization. In order to select cDNA fragments of preferentially 250 ~ 300 bp in length, the library fragments were purified with AMPure XP system (Beckman Coulter, Beverly, USA). 3 µl USER Enzyme (NEB, USA) was used with size-selected, adaptor-ligated cDNA at 37 °C for 15 min, followed by 5 min at 95 °C before PCR. Then, PCR was performed with Phusion High-Fidelity DNA polymerase, Universal PCR primers and Index (X) Primer. At last, PCR products were purified with AMPure XP system and library quality was assessed on the Agilent Bioanalyzer 2100 system [14].

### Clustering and sequencing

The clustering of the index-coded samples was performed with a cBot Cluster Generation System using TruSeq PE Cluster Kit v3-cBot-HS (Illumina). After cluster generation, the library preparations were sequenced on an Illumina Hiseq platform and paired-end reads were generated.

### De novo assembly of short reads and gene annotation

Raw data (raw reads) were firstly processed through in-house perl scripts. Clean data (clean reads) for the JF, PF, SF and M samples were obtained by removing reads containing adapter, reads containing ploy-N and low quality reads from raw data. Simultaneously, Q20, Q30, GC-content and sequence duplication level of the clean data were calculated. All the downstream analyses were based on clean data with high quality. Transcriptome assembly was accomplished based on the left.fq and right.fq using Trinity [18] with min_kmer_cov set to 2 by default, and all other parameters set default. The resulting sequences were named unigenes. The unigenes were annotated by BLASTx searching in NCBI non-redundant (Nr), Swiss-Prot, KEGG, PFAM and KOG and mapped to NCBI Nt database by BLASTn. Functional annotation by Gene Ontology (GO) terms was analyzed by using Blast2GO (http://www.balst2go.org/) software [19]. GO functional classification for unigenes was analyzed using WEGO software [20]. The similarity searches of unigenes were performed using the NCBI-BLAST network server (http://blast.ncbi.nlm.nih.gov/).

### Differential expression genes and GO enrichment analysis

Differential expression analysis between two life-history stages (JF vs PF, PF vs SF and M vs PF) of *D. sinensis* was performed using the DEGseq 2 package. *P*-value was adjusted using *Q-value* [21]. *Q-value* < 0.05 and |log_2_ (fold change)|> 1 were set as the threshold for significantly differential expression [14]. GO enrichment analysis of DEGs was implemented by the GOseq packages based on Wallenius’ non-central hyper-geometric distribution [22].

### Validation of DEGs using Real Time-PCR

Total RNA was extracted using TRIzol reagent (TaKaRa, Dalian, China). The ultramicro-spectrophotometer (MD2000D, Biofuture, UK) was used to assess sample purity and RNA concentration. RNA was reversely transcribed by PrimeScript™ RT reagent Kit (TaKaRa, Dalian, China).

RT-qPCR was performed with 76 genes, which were selected from top 30 up-regulation DEGs in M vs JF, M vs PF and M vs SF. The qPCR primers were designed using Beacon Designer 7.9 (PREMIER Biosoft International, Palo Alto, CA, USA) and listed in Table S3. We tested the amplication efficiency per primer before qPCR validation. *DsimGAPDH* (glyceraldehyde-3-phosphate dehydrogenase) was used as the reference gene and also listed in Table S3. The qPCR was performed in a LightCycler® 96 (Roche Diagnostics Gmbh, Basel, Switzerland) using a mixture of 5.0 μL AceQ qPCR SYBR Green Master Mix (Vazyme, China), 0.2 μL of each primer, 1.0 μL of sample cDNA and 3.6 μL of RNase Free dH_2_O. The amplification step was executed using a degeneration step at 95℃ for 10 min, followed by 40 cycles of 95℃ for 15 s and 60℃ for 60 s. The melting curve was employed to detect a single primer-specific peak, using 93℃ for 30 s and 60℃ for 45 s. All reactions were run in triplicate. The relative quantification results were analyzed using the Ct method (${2}^{{-\Delta \Delta \mathrm{C}}_{\mathrm{T}}}$) [23].

### Statistical analysis

Statistical analysis was executed by SPSS 20.0 software. Significant differences of relative mRNA expression level between two life-history stages (M vs JF, M vs PF and M vs SF) were analyzed using multiple comparisons Turkey (HSD). All data were shown as mean ± SEM in this study.

## Results

### Transcriptome sequencing, assembly and annotation

Among transcriptome sequencing of four life-history stages (JF-juvenile female, PF-parthenogenetic female, SF-sexual female and M-male) of *D. sinensis*, 110437 transcripts were obtained, with a total length of 321269424 bp and an average length of 2909 bp. Moreover, 22996 unigenes were obtained, with a total length of 44512763 bp, an average length of 1936 bp and a N_50_ length of 4265 bp (Table S1). Compared with several common databases through BLASTx program, the most unigenes annotated to Nr database (13512, accounting for 58.75% of the total unigenes), followed by PFAM (10659, accounting for 46.35%) and GO (10659, accounting for 46.35%) (Table S2).

### Homology analysis

**Fig. 1:**
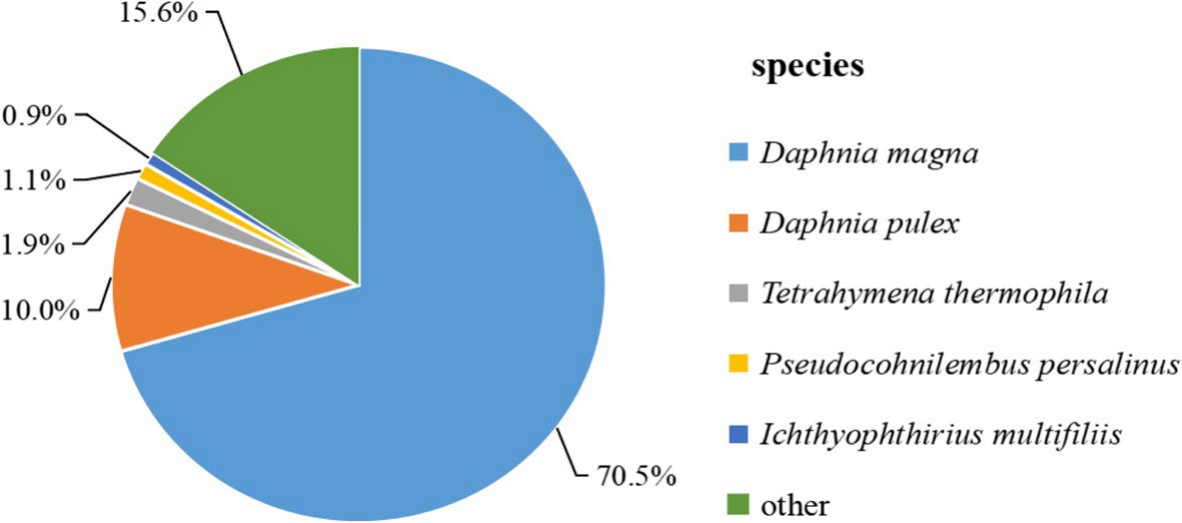
Percentage of homologous hits of *D. sinensis* unigenes to other species

The homologous sequences of *D. sinensis* unigenes were matched in Nr database. The relative species with higher homologous sequences were *D. magna* (70.5%), followed by *D. pulex* (10.0%), *Tetrahymena thermophila* (1.9%), *Pseudocohnilembus persalinus* (1.1%), *Ichthyophthirius multifiliis* (0.9%) and other (15.6%) (Fig. 1).

### Differentially expressed genes

**Fig. 2:**
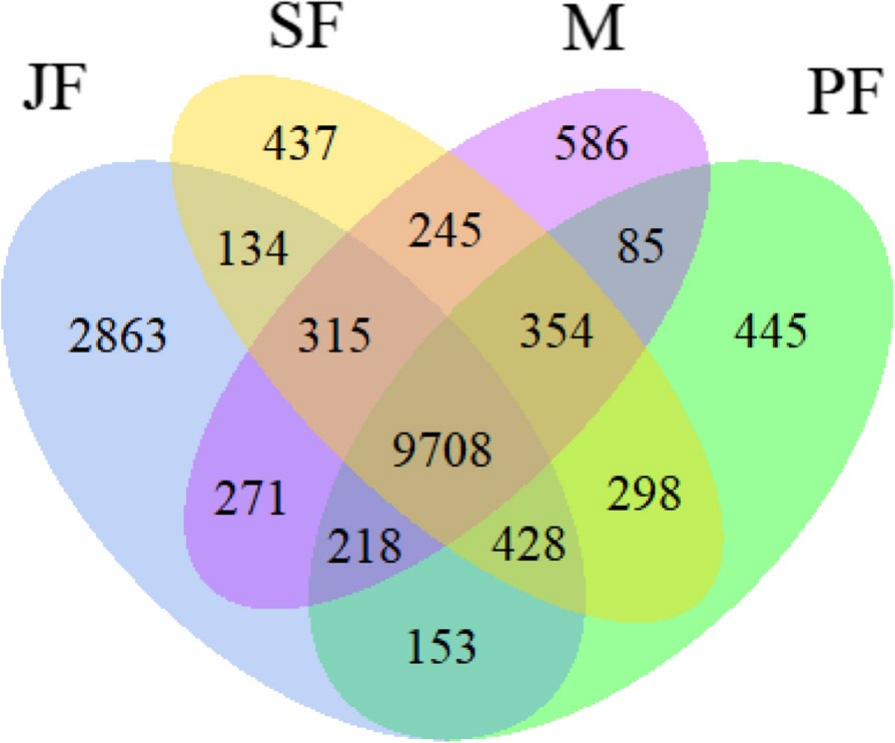
Venn diagram of the number of unigenes with RPKM > 0.3 in four life-history stages (JF, PF, SF and M; RPKM: reads per kilo bases per million mapped)

**Fig. 3:**
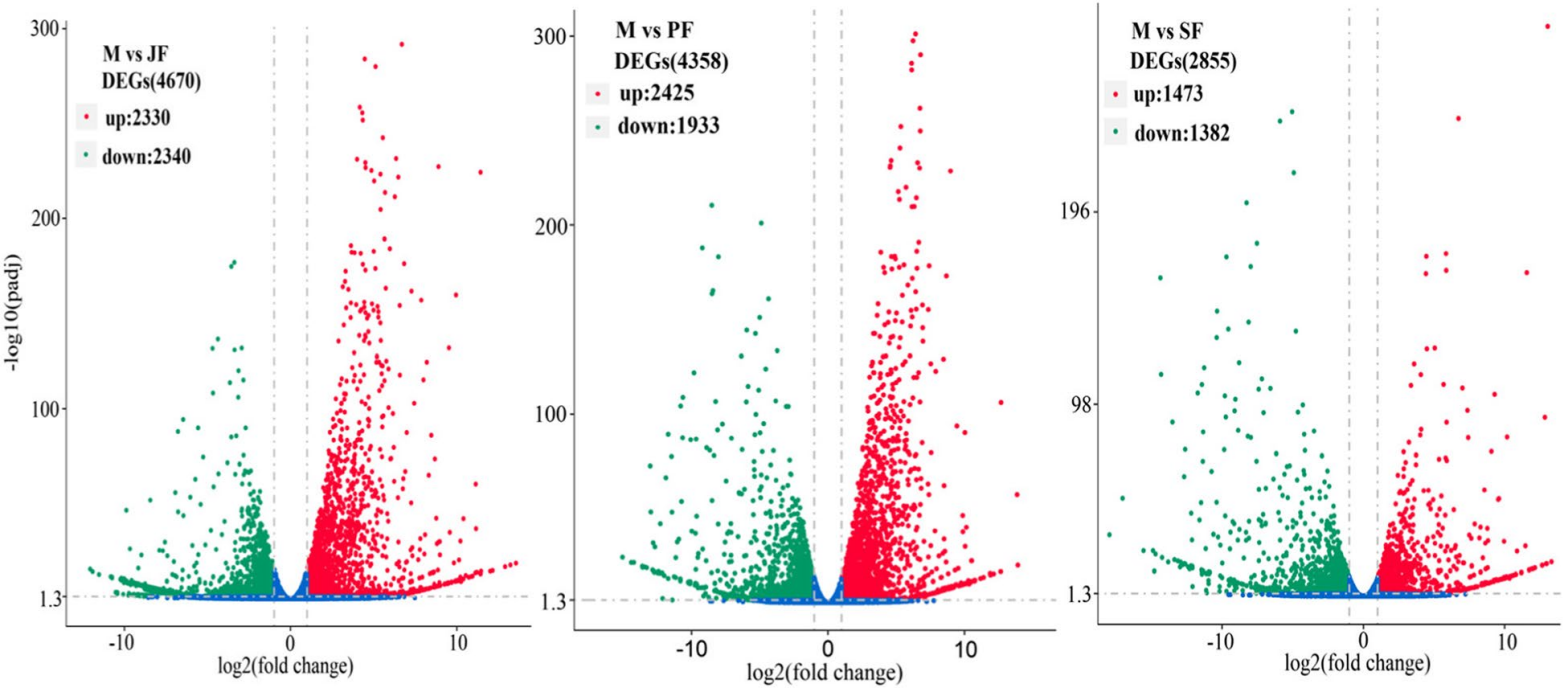
Volcano plot of differentially expressed genes in M vs JF, M vs PF and M vs SF

The number of specific unigenes in JF, PF, SF and M life-history stages were 2863, 445, 437 and 586, respectively. There were 9708 common unigenes in four life-history stages (Fig. 2). The differentially expression genes (DEGs) between the two stages were determined by comparing the genes obtained in male with genes in the three female stages. In differentially expressed genes, the number of up-regulated genes and down-regulated genes were 2230 and 2340 in M vs JF, 2425 and 1933 in M vs PF, and 1473 and 1382 in M vs SF, respectively (Fig. 3).

### Gene ontology annotation

**Fig. 4:**
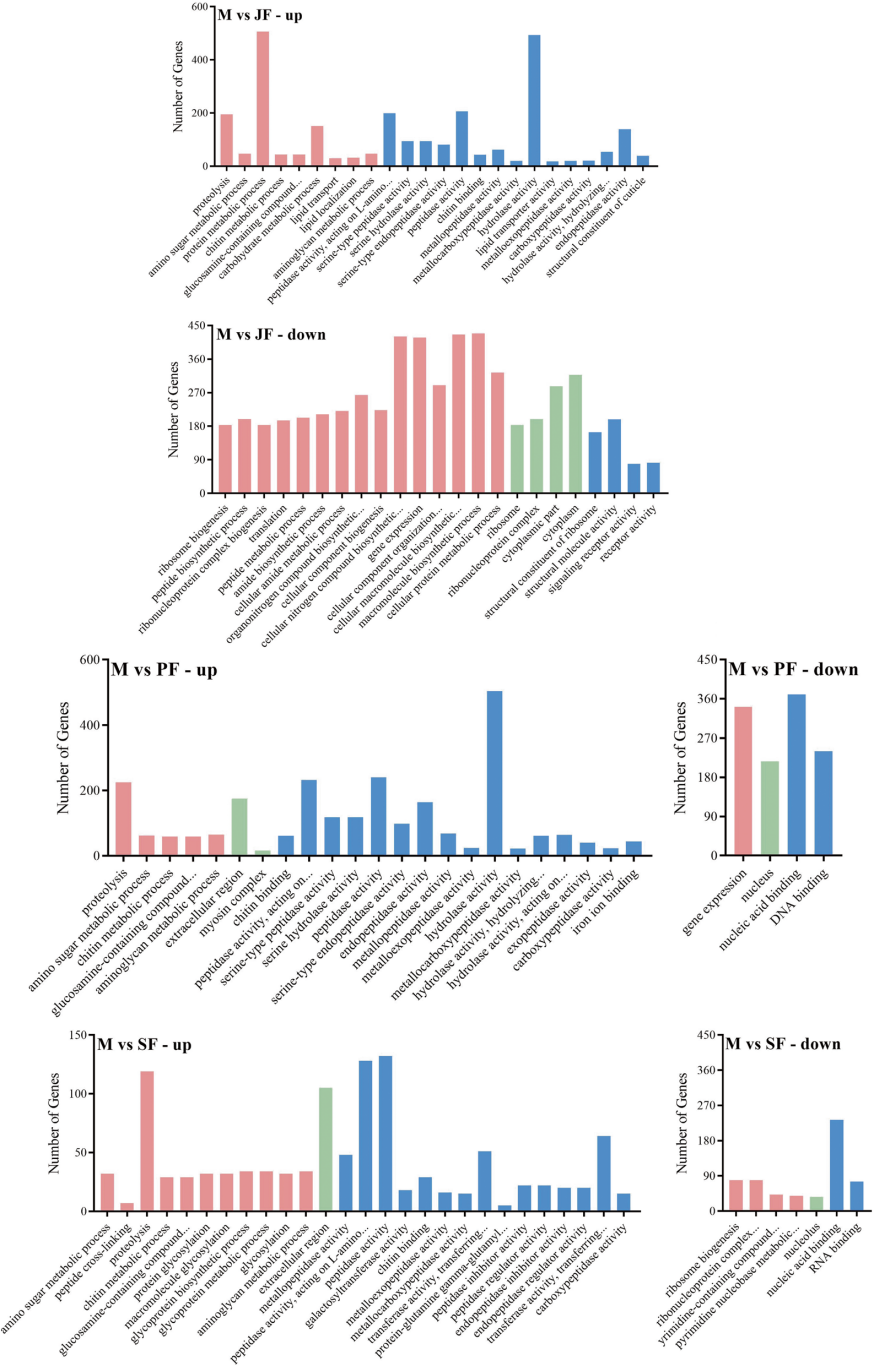
GO enrichment analysis of differentially expressed genes in M vs JF, M vs PF and M vs SF. (red represents biological process, green represents cellular component, blue represents molecular function)

To analyze the functions of these DEGs, we conducted 4670 DEGs in M vs JF, 4358 DEGs in M vs PF and 2855 DEGs in M vs SF by using the GO enrichment system (*Q-value* < 0.05). In M vs JF, up-regulated genes were mainly concentrated in protein metabolic process (506) and hydrolase activity (493), and the down-regulated genes were mainly concentrated in macromolecule biosynthetic process (429), cellular macromolecule biosynthetic process (426), cellular nitrogen compound biosynthetic process (421) and gene expression (418). In M vs PF, the up-regulated genes were mainly concentrated in hydrolase activity (503), and the down-regulated genes were mainly concentrated in nucleic acid binding (370) and gene expression (342). In M vs SF, the up-regulated genes were mainly concentrated in hydrolase activity (132), peptidase activity of acting on L-amino acid peptides (128) and proteolysis (119), and the down-regulated genes were mainly concentrated in nuclear acid binding (233) (Fig. 4).

### Male-biased candidate genes

**Fig. 5:**
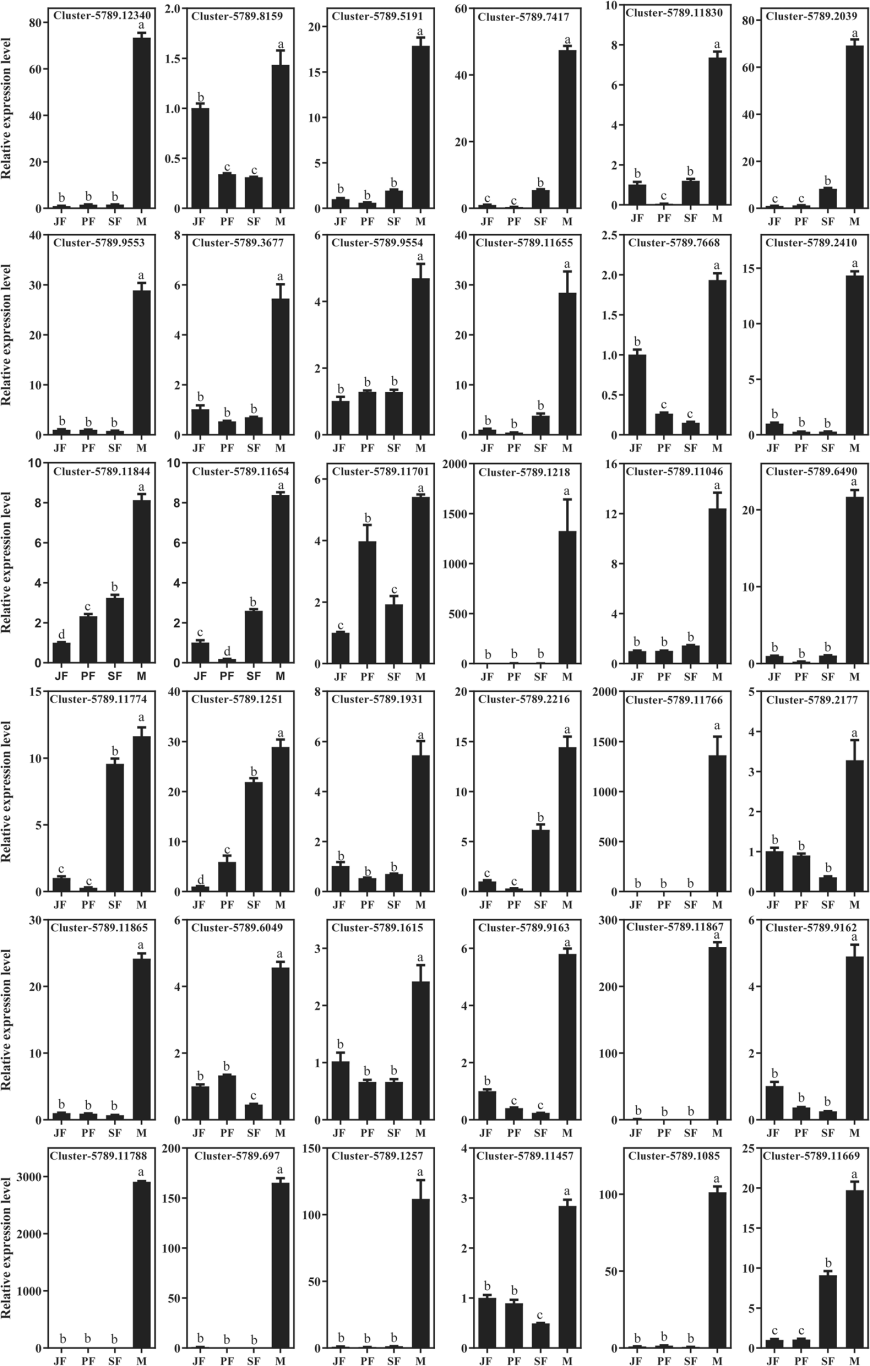
qPCR results of differentially expressed genes related to male-biased genes of *D. sinensis*

In order to screen candidate genes related to male-biased genes of *D. sinensis*, a total of 76 genes were respectively obtained from the top 30 up-regulated DEGs in M vs JF, M vs PF and M vs SF. qPCR analysis showed that the relative expression levels of 36 genes in male (M) were significantly higher than those in the three female stages (JF, PF and SF) (*P* < 0.05), suggesting that these genes may participate in the male sex maintenance of *D. sinensis* (Fig. 5, Table 1). Among them, there are 11 known genes, including one *Doublesex* gene (Cluster-5789.12340), one laminin gene (Cluster-5789.8159), one chitin synthase gene (Cluster-5789.11830), two chitinase genes (Cluster-5789.5191 and Cluster-5789.7417), five trypsin genes (Cluster-5789.9553, Cluster-5789.3677, Cluster-5789.9554, Cluster-5789.11655 and Cluster-5789.7668) and one serine protease gene (Cluster-5789.2039). The other 25 genes were uncharacterized (Table 1).

**Table 1:** Thirty-six differentially expressed genes related to male-biased genes in *D. sinensis*

Gene ID	Nr Description	*q-value*
Cluster-5789.12340	*Doublesex2*	1.31E-175
Cluster-5789.8159	*laminin subunit gamma-3*	3.05E-79
Cluster-5789.5191	*chitinase 5*	1.81E-210
Cluster-5789.7417	*chitinase 8*	1.0536E-284
Cluster-5789.11830	*chitin synthase 2*	2.83E-298
Cluster-5789.2039	*serine protease ami-like*	6.7587E-231
Cluster-5789.9553	*trypsin-7-like protein*	0
Cluster-5789.3677	*putative trypsin-7*	2.1334E-252
Cluster-5789.9554	*putative trypsin-7, partial*	2.1475E-205
Cluster-5789.11655	*trypsin alpha-like*	5.81E-235
Cluster-5789.7668	*trypsin-like isoform X1*	9.1381E-73
Cluster-5789.2410	*uncharacterized protein APZ42_028762*	2.48E-71
Cluster-5789.11844	*uncharacterized protein LOC116923216 isoform X2*	1.00E-77
Cluster-5789.11654	*uncharacterized protein APZ42_030656*	1.96E-46
Cluster-5789.11701	*uncharacterized protein APZ42_020061*	0
Cluster-5789.1218	*uncharacterized protein LOC116919217*	7.5696E-109
Cluster-5789.11046	*uncharacterized protein LOC116919081*	1.26E-220
Cluster-5789.6490	*uncharacterized protein APZ42_017126*	3.32E-214
Cluster-5789.11774	*uncharacterized protein APZ42_015939*	1.63E-292
Cluster-5789.1251	*uncharacterized protein APZ42_031730*	3.07E-256
Cluster-5789.1931	*hypothetical protein DAPPUDRAFT_299805*	6.35E-232
Cluster-5789.2216	*uncharacterized protein APZ42_016906*	3.73E-230
Cluster-5789.11766	*uncharacterized protein APZ42_018760*	7.8031E-166
Cluster-5789.2177	*uncharacterized protein LOC116920378*	2.7322E-165
Cluster-5789.11865	*uncharacterized protein APZ42_014845*	6.39E-185
Cluster-5789.6049	*uncharacterized protein APZ42_018439*	7.96E-127
Cluster-5789.1615	*uncharacterized protein LOC116917111*	2.39E-108
Cluster-5789.9163	*uncharacterized protein LOC116927871*	5.10E-107
Cluster-5789.11867	*uncharacterized protein LOC116922766*	8.63E-104
Cluster-5789.9162	*uncharacterized protein LOC116927870*	1.30E-95
Cluster-5789.11788	*uncharacterized protein LOC116927218*	4.68E-92
Cluster-5789.697	*uncharacterized protein LOC116918656*	1.47E-89
Cluster-5789.1257	*uncharacterized protein APZ42_024939*	6.62E-82
Cluster-5789.11457	*uncharacterized protein LOC116929377 isoform X1*	1.26E-81
Cluster-5789.1085	*uncharacterized protein APZ42_033498*	1.40E-74
Cluster-5789.11669	*uncharacterized protein LOC116935274*	1.6049E-220

**Fig. 6:**
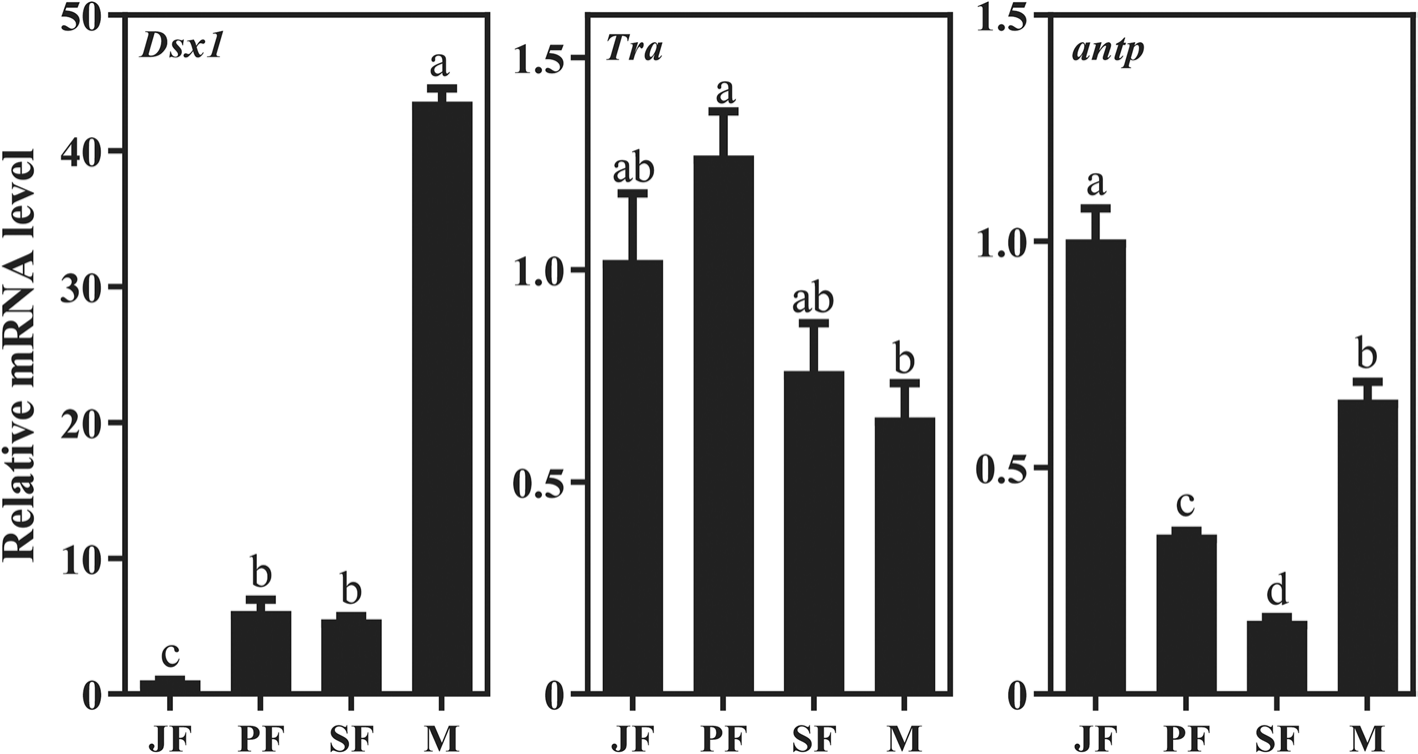
qPCR results of some published genes related to male-biased genes in *D. sinensis.*(*Dsx1* [13], *antp* [12] and *Tra* [14] may be related to male-biased genes in other *Daphnia* species)

In addition, some previous known genes (*Dsx1*, *Tra*, *antp* and DMRT93B) related to male-biased genes in other *Daphnia* species appeared also in the differentially expressed genes of *D. sinensis* (Fig. 6). The expression levels of *Dsx1* in male (M) was significantly (*P* < 0.05) higher than that in the other three stages (JF, PF and SF), and the expression level of *antp* in male (M) was significantly (*P* < 0.05) higher than those in both PF and SF whereas it was significantly lower than that in JF (the abbreviation reference Table S4).

## Discussion

Usually, under worse conditions (such as fish predation, unsuited temperature and photoperiod), *Daphnia* transforms from parthenogenesis to sexual reproduction, producing male and sexual female, which mate and form resting eggs [24–26]. Colbourne et al. (2011) reported the detailed genomes information of *D. pulex*, the amount of genomic and transcriptomic database greatly promoted our understanding of sex-biased genes for *Daphnia*. The related study indicated that male-biased expression is enriched for cuticle and protease function. Moreover, male-biased genes seem to evolve faster than females-biased genes [1, 13]. Based on database, some studies have shown that several genes (e.g. *Dsx*, *antp*, *Tra*, DMRT93B) could play important roles in the male-biased genes of *Daphnia* [4, 27–29]*. Doublesex* (*Dsx*) gene is an important sex regulatory gene, and has been widely studied in Cladocera [30]. Kato et al. (2011) found that knock-out *Dsx1* in male embryos of *D. magna* will lead to the production of female characteristics including ovarian maturation, while ectopic expression of *Dsx1* in female embryos will lead to the life-history of male-like phenotype, and thought that *Dsx1* is a key regulator of male phenotype in *D. magna* [4]. In *D. carinata*, *Dsx1* and *Dsx2* may be also involved in the sex differentiation [31]. *Dsx* contains two conserved domains: one is Dsx/Mab-3 (DM) domain at the N-terminal, and the other is oligomeric domain at the C-terminal [32]. DM-domain plays an important role in the sex maintenance of vertebrates [33, 34]. In this study, the expression of *Doublesex2* (Cluster-5789.12340) in male was significantly (*P* < 0.05) higher than those in the three females (Juvenile female, parthenogenetic female and sexual female), suggesting that *Doublesex2* as male-biased candidate genes may play an important role for male sex maintenance in *D. sinensis*. In *D. magna*, *DMRT93B* is contributed to the differentiation or maintenance of the testis [27]. However, *DMRT93B* did not express in the four life-history stages of *D. sinensis* in this study. The reason for this phenomenon may be that the expression of the same gene is different in different species or different developmental stages.

For other arthropods, the formations of differential morphology of legs and antennae were regulated by the *antp* gene [35, 36]. Our results also showed the expression level of *antp* gene in male was significantly (*P* < 0.05) higher than those in females (parthenogenetic female and sexual female). This suggested that *antp* may be responsible for male sex maintenance in *D. sinensis*. Schwarzenberger and Von Elert (2016) observed also that the *antp* expression level in the first antennae of *D. magna* male adults was significantly higher than that in the first antennae of female adults, and thought that *antp* may be involved in the molecular pathway of inducement to male phenotype of *Daphnia* [28]. Moreover, the expression of *Tra* in *Daphnia carinata* male was significantly higher than those in both parthenogenetic female and sexual female [31], which was also thought to be responsible for male sex maintenance of *Daphnia*. In this study, however, the expression level of *Tra* in *D. sinensis* male was significantly lower than that in parthenogenetic female, indicating that *Tra* may not play an important role in male-biased genes of *D. sinensis*. We speculated that the *Tra* gene maybe play different function in *D. sinensis* compared with *D. carinata*, or some analogous genes with *Tra* may coexist in *D. sinensis.*

Laminin (Ln) is a component of the basement membrane of male and female gonads in the frog *Rana rugosa* [37]. The synthesis of basement membrane is essential for sex differentiation of embryonic mammalian gonads [38]. In this study, the relative expression level of *laminin subunit gamma-3* (Cluster-5789.8159) gene in male was significantly higher than those in the three females. The expression of *laminin alpha 1* (*LAMA1*) in male and female bovine embryos showed sexual dimorphism [39]. Those results implied that *laminin subunit gamma-3* gene may affect male sex differentiation through promoting the life-history of male gonads in *D. sinensis*. In addition, laminin alpha 5 chain is an early molecular marker of sexual differentiation in rat, which may be regulated by the sperm -determining factors [40].

Chitin is the second abundant polysaccharide in nature, and it is the main component of fungal cell wall and exoskeleton of arthropod. Chitin is synthesized by chitin synthase and degraded by chitinase (*Cht*) to maintain the sustainable growth and life-history of organisms [41]. The chitin content in some male insects is significantly higher than that in female insects [42]. In this study, the expression levels of chitin synthase (Cluster-5789.11830) and chitinase (Cluster-5789.5191, Cluster-5789.7417) genes in *D. sinensis* male showed all significantly higher than those in the three females. Same with *Phenacoccus solenopsis*, the expression levels of *Cht 4* and Cht 4–1 in males were significantly higher than those in females [43]. Moreover, the *Cht 4* gene in *Nilaparvata lugens* was only highly expressed in reproductive organs of adult male [44]. Those results suggested that the chitin synthase and chitinase genes play important roles for male sex maintenance in *D. sinensis*. In summary, 36 candicate genes were screened to be responsible for male sex maintenance of *D. sinensis*.

## Conclusions

In this study, transcriptome sequences of the four life-history stages (JF-juvenile female, PF-parthenogenetic female, SF-sexual female and M-male) in *D. sinensis* were investigated, and candidate genes related to male-biased genes were screened (M vs JF, M vs PF and M vs SF)*.* The number of specific unigenes in the four life-history stages (JF, PF, SF and M) were respectively 2863, 445, 437 and 586, with a common unigenes of 9708. Based on DEGs, the number of up-regulated genes and down-regulated genes were 2230 and 2340 in M vs JF, 2425 and 1933 in M vs PF, and 1473 and 1382 in M vs SF, respectively.

The 36 candidate genes related to male-biased genes of *D. sinensis* were obtained through screening from the top 30 up-regulated differentially expressed genes in M vs JF, M vs PF and M vs SF (*P* < 0.05). Among these genes, there are 11 known genes, such as *Doublesex* gene which was involved in sex differentiation of other *Daphnia* species, laminin gene which possibly related to the life-history of male gonads, and two chitinase genes which showed sexually dimorphic expression in *D. sinensis*. In addition, *Dsx*, *Tra* and *antp genes* related to male sex maintenance were also found in differentially expressed genes of four life-history stages in *D. sinensis*. The screening of the candidate genes will provide a reference for the identification of functional genes in *Daphnia* species and the molecular regulation mechanism of sex maintenance in Cladocera. Meanwhile, some results (e.g. DMRT93B) in *D. sinensis* were inconsistent with previous investigations, suggesting that the expression level of functional genes may be related to the life-history stage of organisms, and may be also affected by different *Daphnia* species. However, it was very difficult to find the differences of sex-biased genes among different *Daphnia* species because of the differences in methodology, number of biological replicates, aspects of data analysis and etc. On the other hand, studies on the sex-biased genes (especially male-biased genes) in *Daphnia* species are lack. Therefore, our results will provide some necessary fundamental data for further research of male-biased genes of *Daphnia* in future.

## Supplementary Information

**Additional file 1:** **Table S1.** Assembly analysis of transcriptome from four life-history stages of *Daphnia sinensis*. **Table S2.** Summary statistics on functional annotation of unigenes in *Daphnia sinensis* tanscriptome. **Table S3.** Primers for qPCR genes. **Table S4.** The abbreviation in this study.

## Data Availability

The raw RNA-Seq data used in this study have been deposited in the Nation Center for Biotechnology Information (NCBI) Gene Expression Omnibus (GEO) database under the accession number GSE197943. The web link is “https://www.ncbi.nlm.nih.gov/geo/query/acc.cgi?acc=GSE197943”.

## References

[1] Ellegren H, Parsch J (2007). The evolution of sex-biased genes and sex-biased gene expression. Nat Rev Genet.

[2] Bergero R, Charlesworth D (2009). The evolution of restricted recombination in sex chromosomes. Trends Ecol Evol.

[3] Grath S, Parsch J (2016). Sex-Biased Gene Expression. Annu Rev Genet.

[4] Kato Y, Kobayashi K, Watanabe H, Iguchi T (2011). Environmental sex determination in the branchiopod crustacean *Daphnia magna*: deep conservation of a *Doublesex* gene in the sex-determining pathway. PLoS Genet.

[5] Deng HW (1996). Environmental and genetic control of sexual reproduction in *Daphnia*. Heredity.

[6] Carvalho GR, Hughes RN (1983). The effect of food availability, female culture-density and photoperiod on ephippia production in *Daphnia magna* Straus (Crustacea: Cladocera). Freshwater Biol.

[7] Hobaek A, Larsson P (1990). Sex determination in *Daphnia magna*. Ecology.

[8] Rojas NET, Marins MA, Rocha O (2001). The effect of abiotic factors on the hatching of *Moina micrura* Kürz, 1874 (Crustacea: Cladocera) ephippial eggs. Braz J Biol.

[9] Deng DG, Zhang S, Li YY, Meng XL, Yang W, Li Y, Li XX (2010). Effects of *Microcystis aeruginosa* on population dynamics and sexual reproduction in two *Daphnia* species. J Plankton Res.

[10] Möst M, Oexle S, Marková S, Aidukaite D, Baumgartner L, Stich HB, Wessels M, Martin-Creuzburg D, Spaak P (2015). Population genetic dynamics of an invasion reconstructed from the sediment egg bank. Mol Ecol.

[11] Lampert W, Kinne O (2011). *Daphnia*: development of a model organism in ecology and evolution.

[12] Miner BE, De Meester L, Pfrender ME, Lampert W, Hairston NG (2012). Linking genes to communities and ecosystems: *Daphnia* as an ecogenomic model. P Roy Soc B-Biol Sci.

[13] Colbourne JK, Pfrender ME, Gilbert D, Thomas WK, Tucker A, Oakley TH, Tokishita S, Aerts A, Arnold GJ, Basu MK, Bauer DJ, Cáceres CE, Carmel L, Casola C, Choi JH, Detter JC, Dong Q, Dusheyko S, Eads BD, Fröhlich T, Geiler-Samerotte KA, Gerlach D, Hatcher P, Jogdeo S, Krijgsveld J, Kriventseva EV, Kültz D, Laforsch C, Lindquist E, Lopez J, Manak JR, Muller J, Pangilinan J, Patwardhan RP, Pitluck S, Pritham EJ, Rechtsteiner A, Rho M, Rogozin IB, Sakarya O, Salamov A, Schaack S, Shapiro H, Shiga Y, Skalitzky C, Smith Z, Souvorov A, Sung W, Tang Z, Tsuchiya D, Tu H, Vos H, Wang M, Wolf YI, Yamagata H, Yamada T, Ye Y, Shaw JR, Andrews J, Crease TJ, Tang H, Lucas SM, Robertson HM, Bork P, Koonin EV, Zdobnov EM, Grigoriev IV, Lynch M, Boore JL (2011). The ecoresponsive genome of *Daphnia pulex*. Science.

[14] Zhang YN, Zhu XY, Wang WP, Wang Y, Wang L, Xu XX, Zhang K, Deng DG (2016). Reproductive switching analysis of *Daphnia similoides* between sexual female and parthenogenetic female by transcriptome comparison. Sci Rep.

[15] Huylmans AK, LópezEzquerra A, Parsch J, Cordellier M. De Novo Transcriptome Assembly and Sex-Biased Gene Expression in the Cyclical Parthenogenetic *Daphnia galeata*. Genome Biol Evol. 2016;8(10):3120–39. 10.1093/gbe/evw221 PMC5174735 27604882

[16] Molinier C, Reisser CMO, Fields P, Ségard A, Galimov Y, Haag CR. Identification of General Patterns of Sex-Biased Expression in Daphnia, a Genus with Environmental Sex Determination. G3 (Bethesda). 2018;8(5):1523–33. 10.1534/g3.118.200174 PMC5940145 29535148

[17] Jia J, Dong C, Han M, Ma S, Chen W, Dou J, Feng C, Liu X (2022). Multi-omics perspective on studying reproductive biology in *Daphnia sinensis*. Genomics.

[18] Grabherr MG, Haas BJ, Yassour M, Levin JZ, Thompson DA, Amit I, Adiconis X, Fan L, Raychowdhury R, Zeng Q, Chen Z, Mauceli E, Hacohen N, Gnirke A, Rhind N, di Palma F, Birren BW, Nusbaum C, Lindblad-Toh K, Friedman N, Regev A (2011). Full-length transcriptome assembly from RNA-Seq data without a reference genome. Nat Biotechnol.

[19] Conesa A, Götz S, García-Gómez JM, Terol J, Talón M, Robles M (2005). Blast2GO: a universal tool for annotation, visualization and analysis in functional genomics research. Bioinformatics.

[20] Ye J, Fang L, Zheng H, Zhang Y, Chen J, Zhang Z, Wang J, Li S, Li R, Bolund L, Wang J (2006). WEGO: a web tool for plotting GO annotations. Nucleic Acids Res.

[21] Storey JD (2003). The positive false discovery rate: a Bayesian interpretation and the *q*-value. Ann Stat.

[22] Young MD, Wakefield MJ, Smyth GK, Oshlack A (2010). Gene ontology analysis for RNA-seq: accounting for selection bias. Genome Biol.

[23] Livak KJ, Schmittgen TD (2001). Analysis of relative gene expression data using real-time quantitative PCR and the 2^-△△CT^ method. Methods.

[24] Camp AA, Haeba MH, LeBlanc GA (2019). Complementary roles of photoperiod and temperature in environmental sex determination in *Daphnia* spp. J Exp Biol.

[25] Eads BD, Andrews J, Colbourne JK (2008). Ecological genomics in *Daphnia*: stress responses and environmental sex determination. Heredity.

[26] Gust KA, Kennedy AJ, Laird JG, Wilbanks MS, Barker ND, Guan X, Melby NL, Burgoon LD, Kjelland ME, Swannack TM (2019). Different as night and day: behavioural and life history responses to varied photoperiods in *Daphnia magna*. Mol Ecol.

[27] Kato Y, Kobayashi K, Oda S, Colbourn JK, Tatarazako N, Watanabe H, Iguchi T (2008). Molecular cloning and sexually dimoraphic expression of DM-domain genes in *Daphnia magna*. Genomics.

[28] Schwarzenberger A, Von Elert E (2016). What makes a man a man? prenatal antennapedia expression is involved in the formation of the male phenotype in *Daphnia*. Dev Genes Evol.

[29] Chen P, Xu SL, Zhou W, Guo XG, Wang CL, Wang DL, Zhao YL (2014). Cloning and expression analysis of a transformer gene in *Daphnia pulex* during different reproduction stages. Anim Reprod Sci.

[30] Burtis KC, Baker BS (1989). *Drosophila doublesex* gene controls somatic sexual differentiation by producing alternatively spliced mRNAs encoding related sex-specific polypeptides. Cell.

[31] Kong L, Lv WW, Huang YH, Liu ZQ, Yang Y, Zhao YL (2015). Cloning, expression and localization of the *Daphnia carinata transformer* gene *DcarTra* during different reproductive stages. Gene.

[32] Bayrer JR, Zhang W, Weiss MA (2005). Dimerization of *doublesex* is mediated by a cryptic ubiquitin-associated domain fold: implications for sex-specific gene regulation. J Biol Chem.

[33] Raymond CS, Shamu CE, Shen MM, Seifert KJ, Hirsch B, Hodgkin J, Zarkower D (1998). Evidence for evolutionary conservation of sex-determining genes. Nature.

[34] Matsuda M, Nagahama Y, Shinomiya A, Sato T, Matsuda C, Kobayashi T, Morrey CE, Shibata N, Asakawa S, Shimizu N, Hori H, Hamaguchi S, Sakaizumi M (2002). DMY is a Y-specific DM-domain gene required for male development in the medaka fish. Nature.

[35] Struhl G (1981). A homoeotic mutation transforming leg to antenna in *Drosophila*. Nature.

[36] Khadjeh S, Turetzek N, Pechmann M, Schwager EE, Wimmer EA, Damen WG, Prpic NM (2012). Divergent role of the Hox gene *Antennapedia* in spiders is responsible for the convergent evolution of abdominal limb repression. P Natl Acad Sci USA.

[37] Saotome K, Isomura T, Seki T, Nakamura Y, Nakamura M (2010). Structural changes in gonadal basement membranes during sex differentiation in the frog *Rana rugosa*. J Exp Zool Part A.

[38] Pelliniemi LJ, Fröjdman K, Sundström J, Pöllänen P, Kuopio T (1998). Cellular and molecular changes during sex differentiation of embryonic mammalian gonads. J Exp Zool.

[39] Bermejo-Alvarez P, Rizos D, Lonergan P, Gutierrez-Adan A (2011). Transcriptional sexual dimorphism in elongating bovine embryos: implications for XCI and sex determination genes. Reproduction.

[40] Fröjdman K, Miner JH, Sanes JR, Pelliniemi LJ, Virtanen I (1999). Sex-specific localization of *laminin alpha 5 chain* in the differentiating rat testis and ovary. Differentiation.

[41] Khoushab F, Yamabhai M (2010). Chitin research revisited. Mar Drugs.

[42] Kulma M, Kouřimská L, Plachý V, Božik M, Adámková A, Vrabec V. Effect of sex on the nutritional value of house cricket, *Acheta domestica* L. Food Chem. 2019;272:267–72. 10.1016/j.foodchem.2018.08.049 30309543

[43] Omar MAA, Ao Y, Li M, He K, Xu L, Tong H, Jiang M, Li F. The functional difference of eight chitinase genes between male and female of the cotton mealybug, *Phenacoccus solenopsis*. Insect Mol Biol. 2019;28(4):550–67. 10.1111/imb.12572 30739379

[44] Xi Y, Pan PL, Ye YX, Yu B, Xu HJ, Zhang CX (2015). Chitinase-like gene family in the brown planthopper, Nilaparvata lugens. Insect Mol Biol.

