# Supplementary material for: Transcriptome analysis of different life-history stages and screening of male-biased genes in Daphnia sinensis
Source: BMC Genomics. 2022 Aug 13;23:589. doi: 10.1186/s12864-022-08824-x (PMC9375365; doi:10.1186/s12864-022-08824-x)
Supplement: Supplementary file 1 — Additional file 1: Table S1. Assembly analysis of transcriptome from four life-history stages of Daphnia sinensis. Table S2. Summary statistics on functional annotation of unigenes in Daphnia sinensis tanscriptome. Table S3. Primers for qPCR genes. Table S4. The abbreviation in this study. [file 12864_2022_8824_MOESM1_ESM.docx]

**Supplementary Material (SM)**

**Table S1** Assembly analysis of transcriptome from four life-history stages of *Daphnia sinensis*

|  | Transcripts | Unigenes |
| --- | --- | --- |
| 200-500bp | 18137 | 6898 |
| 500-1000 bp | 17791 | 6040 |
| 1k-2000 bp | 20536 | 3619 |
| >2000 bp | 53973 | 6439 |
| Total number | 110437 | 22996 |
| Total length | 321269424 | 44512763 |
| Mean length | 2909 | 1936 |
| N_50_ length | 5000 | 4265 |

**Table S2** Summary statistics on functional annotation of unigenes in *Daphnia sinensis* tanscriptome

|  | Number of Genes | Percentage (%) |
| --- | --- | --- |
| Nr | 13512 | 58.75 |
| Nt | 3957 | 17.20 |
| KEGG | 6211 | 27.00 |
| Swiss-Prot | 9354 | 40.67 |
| PFAM | 10659 | 46.35 |
| KOG | 6472 | 28.14 |
| GO | 10659 | 46.35 |

**Table S3** Primers for qPCR genes

| Application | Primer name | Primer sequence |
| --- | --- | --- |
| Male-biased gene | Cluster-5789.12340-F | ATAGCATTGAATCCTGTGAAG |
|  | Cluster-5789.12340-R | CATACCTTGTGACCTCTGA |
|  | Cluster-5789.8159-F | AGAAGACTTACCATCGCATA |
|  | Cluster-5789.8159-R | TCCATCGTGAAGATTGAGA |
|  | Cluster-5789.5191-F | ATCCGTTATCTTCTTACTCGTA |
|  | Cluster-5789.5191-R | ATCATCATAGCCAATCCATTG |
|  | Cluster-5789.7417-F | AGGATCAGCGTAGTAAGC |
|  | Cluster-5789.7417-R | CATCAGCATCAACCACAG |
|  | Cluster-5789.11830-F | CTCCATCGCTTCCTGTTA |
|  | Cluster-5789.11830-R | GCATCGTTCGTTCATCAA |
|  | Cluster-5789.2039-F | AGACACTGACTCCAATCTC |
|  | Cluster-5789.2039-R | CCTGACACTGCTTGACTA |
|  | Cluster-5789.9553-F | GTGGTGTCGTAGTTCTCA |
|  | Cluster-5789.9553-R | AATGTCAGGTTTGTTCAATCT |
|  | Cluster-5789.3677-F | TACTGGCTGAAGATGTCTC |
|  | Cluster-5789.3677-R | ATGGCGATGAACACAATG |
|  | Cluster-5789.9554-F | GACACTTGCCAGGTTATATG |
|  | Cluster-5789.9554-R | TAGCCAAAGGAGGTGATAC |
|  | Cluster-5789.11655-F | GTAATCGCCGCATTGAAT |
|  | Cluster-5789.11655-R | TGATGTGCCTGGTATTGT |
|  | Cluster-5789.7668-F | CCAATACGGAACTTCATTCAT |
|  | Cluster-5789.7668-R | GGTAATGCCAACTTGAACA |
|  | Cluster-5789.2410-F | GTATCGTCATTGTCATTAGCA |
|  | Cluster-5789.2410-R | CGTCACTTCTCACTCACT |
|  | Cluster-5789.11844-F | TCGTAGCCTTGAGGATTC |
|  | Cluster-5789.11844-R | GCACAACTAACAGATACCATT |
|  | Cluster-5789.11654-F | AGCGACCAATACGAATCT |
|  | Cluster-5789.11654-R | CAGACCGTTGTAACCAATC |
|  | Cluster-5789.11701-F | TTCAGCAGCATCAACAAC |
|  | Cluster-5789.11701-R | AGCAATCAGACGAACAGA |
|  | Cluster-5789.1218-F | CGAGATGACGAAGAAGGA |
|  | Cluster-5789.1218-R | TTGAATTGGCTGAGTTATACC |
|  | Cluster-5789.11046-F | GAAGTTACCTCACCTCACA |
|  | Cluster-5789.11046-R | GATGAATCTGATGAATCGGTTA |
|  | Cluster-5789.6490-F | TTCTCCACATCGCCATAA |
|  | Cluster-5789.6490-R | TGTTAGCATCACCGTCAT |
|  | Cluster-5789.11774-F | TATGCGTTCTGTGGAAGT |
|  | Cluster-5789.11774-R | CTGGTATTGCTGGAGTAGT |
|  | Cluster-5789.1251-F | GATGCCGAACTCTATGGA |
|  | Cluster-5789.1251-R | GTCAATGATACCGTCGTAAC |
|  | Cluster-5789.1931-F | AGGATACCTCATCGTTCAAT |
|  | Cluster-5789.1931-R | ATCAATATAGACCGCAATTCG |
|  | Cluster-5789.2216-F | GGATGAACCTGGTGAGAT |
|  | Cluster-5789.2216-R | AGAGGCATAGTTGGTGTAG |
|  | Cluster-5789.11766-F | CAGCAACAGAACCAACAA |
|  | Cluster-5789.11766-R | GTCGTAGAGGAGGAACAG |
|  | Cluster-5789.2177-F | TTGGAGAGTAAGGATTCTATGT |
|  | Cluster-5789.2177-R | TCATTCATCAGCAGGACTTA |
|  | Cluster-5789.11865-F | CATGTTGAATGAAGAGGAGAG |
|  | Cluster-5789.11865-R | GTGTGGTTATCTGGAGGAA |
|  | Cluster-5789.6049-F | GTATGTGATGTGCTGGAATT |
|  | Cluster-5789.6049-R | AGTGGCTAATGCTCTGTAT |
|  | Cluster-5789.1615-F | CGCCAATGGAGTAACAAC |
|  | Cluster-5789.1615-R | TAAGACCACCGTGAAGAC |
|  | Cluster-5789.9163-F | ACTATCTCCAATCAGCACTT |
|  | Cluster-5789.9163-R | CACGAGCACAACATACAC |
|  | Cluster-5789.11867-F | TTCTTGTCGTCGGATTCA |
|  | Cluster-5789.11867-R | GGCAGTTGGAATTACATCAT |
|  | Cluster-5789.9162-F | ATTGAACATCGTCCTGGTA |
|  | Cluster-5789.9162-R | TTCGGTCTTAATTACTTGGTTAG |
|  | Cluster-5789.11788-F | CAGGACTTGGAGTGGATT |
|  | Cluster-5789.11788-R | GGAGAGGAGGAATGGTTC |
|  | Cluster-5789.697-F | CACAACAGCCTCTATTGC |
|  | Cluster-5789.697-R | TCGTACACTTGACCTTCC |
|  | Cluster-5789.1257-F | CCGCCAACAGTTCATTAG |
|  | Cluster-5789.1257-R | TCGCAGACAGTCAATTCT |
|  | Cluster-5789.11457-F | CACCACTCTACCGCTATT |
|  | Cluster-5789.11457-R | TGTTCTGATAACTGACTCCA |
|  | Cluster-5789.1085-F | GGAGGAACGATGGTATCTT |
|  | Cluster-5789.1085-R | AGTGGTTGTTGGAGTAGG |
|  | Cluster-5789.11669-F | CAATCAACATTCACTCTGCTA |
|  | Cluster-5789.11669-R | GCTCCTCTTCAACTTCAATC |
| Reference gene | GAPDH-qF | AGGAAGCCAGTTATGATGA |
|  | GAPDH-qR | AAGTCTTGTTGAGAGCAATG |

**Table S4** The abbreviation in this study

| Abbreviation | Definition |
| --- | --- |
| JF | juvenile female |
| PF | parthenogenetic female |
| SF | sexual female |
| M | male |
| DEGs | differentially expressed genes |
| GO | Gene Ontology |
| *Dsx* | *Doublesex* |
| *Tra* | *Transformer* |
| *antp* | *antennapedia* |
| *Ftz-F1* | Fushi tarazu factor-1 |
| JHAMT | juvenile hormone acid O-methyltransferase |
| MF | methyl farnesoate; |
| RT-qPCR | Quantitative real-time polymerase chain reaction |
| *DsimGAPDH* | glyceraldehyde-3-phosphate dehydrogenase; |
| *Ln* | Laminin |
| *LAMA1* | laminin alpha 1 |
| *Cht* | chitinase |
